# Host proteins associated with strong neutralizing SARS-CoV-2 antibody responses in a South African cohort

**DOI:** 10.1038/s43856-026-01427-7

**Published:** 2026-02-13

**Authors:** Afrah Khairallah, Zesuliwe Jule, Alice Piller, Mallory Bernstein, Kajal Reedoy, Yashica Ganga, Sashkia R. Balla, Prudence Kgagudi, Bernadett I. Gosnell, Farina Karim, Mahomed-Yunus S. Moosa, Thumbi Ndung’u, Thandeka Moyo-Gwete, Penny L. Moore, Khadija Khan, Alex Sigal

**Affiliations:** 1 Africa Health Research Institute and University of KwaZulu-Natal, Durban, South Africa; 2 National Institute for Communicable Diseases of the National Health Laboratory Service, Johannesburg, South Africa; 3 Department of Infectious Diseases, Nelson R. Mandela School of Clinical Medicine, University of KwaZulu-Natal, Durban, South Africa; 4 HIV Pathogenesis Programme, University of KwaZulu-Natal, Durban, South Africa; 5 Ragon Institute of Massachusetts General Hospital, Massachusetts Institute of Technology and Harvard University, Cambridge, MA USA; 6 Division of Infection and Immunity, University College London, London, UK; 7 SAMRC Antibody Immunity Research Unit, School of Pathology, Faculty of Health Sciences, University of the Witwatersrand, Johannesburg, South Africa; 8 The Lautenberg Center for Immunology and Cancer Research, Faculty of Medicine, Hebrew University of Jerusalem, Jerusalem, Israel

**Keywords:** Antibodies, SARS-CoV-2

## Abstract

**Background:**

Severe Covid-19 leads to higher neutralizing antibody levels, a key correlate of protection. However, the host proteins associated with this response have not been fully characterized. We asked which proteins in the blood plasma associate with neutralization, anti-spike antibody levels, and disease severity in a South African cohort upon first SARS-CoV-2 exposure.

**Methods:**

We used a longitudinal observational cohort design to collect blood at 6 days (acute infection) and 32 days (convalescence) post-diagnosis. We performed SomaScan proteomics on acute blood plasma and measured SARS-CoV-2 plasma neutralization capacity and anti-spike antibody levels in convalescent plasma. Disease severity was scored based on requirement for supplemental oxygen and was mild to moderate (no critically ill participants).

**Results:**

We find differentially expressed proteins associating with neutralization, anti-spike antibody levels, and disease severity, with strong overlap between proteins associated with neutralization and spike binding, and moderate overlap between neutralization and disease severity. High neutralizers, regardless of requirement for supplemental oxygen, are found to have risk factors and markers for being more ill compared to low neutralizers. We can reasonably predict who becomes a high neutralizer based on individual proteins. The best predictor for neutralization is HSPA8, known to bind viral proteins and cross-present extracellular antigens. The strongest associated pathway is fatty acid metabolism, whose inhibition results in suppression of viral replication.

**Conclusions:**

These results show that host proteins and pathways involved early in SARS-CoV-2 infection associate with neutralizing antibody levels elicited by the infection at convalescence.

## Plain Language Summary

The immune response to SARS-CoV-2 infection leads to the production of antibodies that prevent the virus from infecting cells. This protects people from future infections with the same virus. People differ in their ability to make antibody responses after infection. The source of this variability, in terms of differences in expressed proteins, remains poorly understood. Here, we categorize South African study participants who were infected with SARS-CoV-2 for the first time into those with high and low antibody responses and link this with differences in specific proteins close to the time of infection. We find that proteins and pathways which play a role in SARS-CoV-2 infection also associate with stronger antibody responses.

## Abstract (web summary)

Khairallah et al., investigate the host proteins associated with a strong neutralizing antibody response to SARS-CoV-2. High neutralizing antibody responses to SARS-CoV-2 are associated with specific protein expression patterns, especially HSPA8 and pathways like fatty acid metabolism, which can predict immune strength and correlate with disease severity.

## Introduction

Acute viral infections elicit a neutralizing antibody response, which protects convalescent individuals from symptomatic disease upon re-infection with the same viral serotype^1^. In SARS-CoV-2, neutralizing antibodies prevent the viral spike protein from accessing the angiotensin-converting enzyme 2 (ACE2) viral receptor^2^. Until the neutralizing antibody evasive Omicron variant emerged, re-infection was rare^3^. The degree of protection from symptomatic infection, which is mediated by vaccines, also strongly correlates with neutralizing antibody levels^4–7^. Neutralizing antibodies, a subset of antibodies to SARS-CoV-2 spike, need to bind to specific epitopes either on the spike receptor binding domain (RBD)^2,8^ or the N-terminal domain (NTD)^9^ to effectively interfere with cell entry. Among neutralizing antibodies, different potencies are observed based on binding site and affinity^10^, which may be increased by affinity maturation^11,12^.

Higher disease severity is associated with higher neutralizing antibodies after SARS-CoV-2 infection^13–24^. However, the emergence of the neutralizing antibody response in severe disease may be delayed^14^. Severe Covid-19 is usually caused by acute respiratory distress syndrome (ARDS). Here, inflammation in the lower respiratory tract leads to inflammatory vascular leak, impairing gas exchange^25,26^. Therefore, a key measure of disease severity is hypoxia and the resulting requirement for supplemental oxygen^27^. Additional correlates of more severe disease include an elevated neutrophil to lymphocyte ratio (NLR)^28–33^ and low lymphocyte concentrations (lymphopenia)^32,34–37^ in the blood. Factors predisposing to higher disease severity and mortality in Covid-19 include older age, male sex, diabetes, hypertension, and sometimes HIV co-infection^28,38^.

Covid-19 disease severity level is usually scored by whether supplemental oxygen is administered, the mode of oxygen administration, and additional evidence for severe disease, such as admission to an intensive care unit (ICU). Highest on the scale of disease severity is Covid-19 related death^27^. Despite the strong association between the strength of the neutralizing antibody response and disease severity^13–24^, there is extensive heterogeneity in neutralization levels within each severity category. For example, hospitalized people who are not critically ill show neutralizing antibody levels that range from below the limit of detection to levels observed in critically ill individuals^13^. What determines the heterogeneity in neutralizing capacity between people with a similar level of disease severity is not well understood.

Here, we investigated which proteins early in infection associate with neutralization, anti-spike antibody levels, and disease severity in a South African cohort. We performed SomaScan proteomics of the blood plasma soon after infection and determined the cellular proteins associated with stronger neutralizing and anti-spike antibody responses at convalescence, or for disease severity, maximal severity before discharge. To avoid confounding the results with different re-infection and vaccination histories^39^, we selected individuals from our previously described cohort^28^ who were infected by ancestral SARS-CoV-2 in the first Covid-19 infection wave in South Africa. This was before vaccination was available and before SARS-CoV-2 variants arose. Given the rarity of re-infection pre-Omicron^40^, especially within the same infection wave, the infections we studied here very likely represent the first exposures to SARS-CoV-2. We also explored the relationship between differentially expressed proteins in high versus low neutralizers and differentially expressed proteins between participants with high versus low anti-spike binding antibodies, as well as differentially expressed proteins that associate with disease severity. We note that we did not focus this study on obtaining proteomic signatures for severe Covid-19 disease. This is because our cohort differs from those used in many of the proteomic studies on Covid-19 disease severity^41–44^ in that it does not contain critically ill individuals (e.g., none of the participants died, were ventilated, or admitted to the ICU).

We find that there is extensive overlap between proteins associated with neutralization and anti-spike antibodies and more limited overlap in proteins associated with neutralization versus higher disease severity. Despite the limited overlap, the proteins found to be associated with neutralization are dependent on disease severity, and excluding participants who require supplemental oxygen from the analysis of high versus low neutralizers leads to the loss of almost all significantly differentially expressed proteins. The level of heat shock protein family A member 8 (HSPA8, also called Hsc70 and Hsp73), a member of the heat shock protein 70 family of chaperone proteins, which has multiple functions including antigen presentation on MHC class II molecules^45,46^ and cross-presentation of extracellular antigens to antigen-presenting cells^47^, emerges as one important factor associated with a stronger infection-elicited neutralizing antibody response.

## Methods

### Informed consent and ethical statement

This was a prospective observational study with longitudinal sample collection. The study protocol was approved by the Biomedical Research Ethics Committee at the University of KwaZulu-Natal (reference BREC/00001275/2020). Adult patients (>18 years old) presenting at King Edward VIII, Inkosi Albert Luthuli Central, or Clairwood hospitals in Durban, South Africa, between study inception in June 2020 to October 2020 (end of ancestral SARS-CoV-2 infection wave), who were diagnosed by qPCR to be SARS-CoV-2 positive as part of their clinical workup, and were able to provide informed consent, were eligible for study enrollment. Sample size was not predetermined, and all eligible participants were enrolled. Written informed consent was obtained for all enrolled participants. Only participants with at least one additional sample taken after the enrollment sample were included in the analysis, and one participant was excluded from the analysis because of severe immunocompromise because of advanced HIV disease. Blood samples were obtained at each study visit, and blood plasma from the enrollment sample (acute infection) was used for proteomics, and blood plasma from the convalescent visit (sample closest to 1 month-post diagnosis if multiple post-enrollment samples were available for a given participant) was used for live virus neutralization assays and measurement of anti-spike antibody levels. Participants were reimbursed for each visit based on time, inconvenience, and expenses as approved in the protocol. Disease severity was determined by whether a participant required supplemental oxygen at any time during the tracking period from enrollment to first outpatient or, if lost to follow-up, last inpatient visit. Duration of the tracking period was a median of 14 days (IQR 7-14 days). No participants required high-flow oxygen or ventilation. The requirement for supplemental oxygen, as well as presence of hypertension and diabetes (self-report), were captured using REDCap version 11.1.29.

### Calculation of tracking period for disease severity

Study visits occurred at weekly intervals from enrollment for the first month, where the first study visit was at enrollment. The tracking period for disease severity was set to approximately cover the period from enrollment to discharge, calculated as the time between enrollment and the first outpatient visit. If the participant was lost to follow-up post-discharge, the tracking period was the time between enrollment and the last inpatient visit. Given that the usual quarantine period was 10 days before de-isolation and that enrollment occurred a median of 6 days post-hospital admission, discharge most often occurred between the second and third study visits, and the tracking period was most often enrollment to the third study visit (14 days post-enrollment).

### Clinical laboratory testing

Lymphocyte, neutrophil, and CD4 T cell concentrations, and HIV viral load quantification were performed from a 4 mL EDTA tube of blood at an accredited diagnostic laboratory: Ampath for cell concentrations and Molecular Diagnostic Services for HIV viral load, both based in Durban, South Africa.

### Cells

The H1299-E3 cell line (H1299 originally from ATCC as CRL-5803 and engineered to stably express the ACE2 receptor) was propagated in growth medium consisting of complete Roswell Park Memorial Institute (RPMI) (1640 Sigma-Aldrich, R6504) medium with 10% fetal bovine serum containing 10 mM HEPES, 1 mM sodium pyruvate, 2 mM l-glutamine, and 0.1 mM nonessential amino acids.

### Live virus neutralization assay (focus reduction neutralization assay)

For all neutralization assays, viral input was 100 focus-forming units per well of a 96-well plate. H1299-E3 cells were plated in a 96-well plate (Corning) at 30,000 cells per well 1 day pre-infection. Plasma was separated from EDTA-anticoagulated blood by centrifugation at 500 × *g* for 10 min and stored at −80 °C. Aliquots of plasma samples were heat-inactivated at 56 °C for 30 min and clarified by centrifugation at 10,000 × *g* for 5 min. Virus stocks were added to serially diluted plasma in a 96-well plate (Corning), and plasma–virus mixtures were incubated for 1 h at 37 °C, 5% CO_2_. Cells were infected with 100 μL of the plasma–virus mixtures for 1 h, then 100 μL of a 1X RPMI 1640 (Sigma-Aldrich, R6504), 1.5% carboxymethylcellulose (Sigma-Aldrich, C4888) overlay was added without removing the inoculum. Cells were fixed 20 h post-infection using 4% PFA (Sigma-Aldrich, P6148) for 20 min. Foci were stained with a rabbit anti-spike monoclonal antibody (BS-R2B12, GenScript A02058) at 0.5 μg/mL in a permeabilization buffer containing 0.1% saponin (Sigma-Aldrich, S7900), 0.1% BSA (Biowest, P6154) and 0.05% Tween-20 (Sigma-Aldrich, P9416) in PBS for 2 h at room temperature with shaking, then washed with wash buffer containing 0.05% Tween-20 in PBS. Secondary goat anti-rabbit HRP conjugated antibody (Abcam ab205718) was added at 1 μg/mL and incubated for 2 h at room temperature with shaking. TrueBlue peroxidase substrate (SeraCare 5510-0030) was then added at 50 μL per well and incubated for 20 min at room temperature. Plates were imaged in an ImmunoSpot Ultra-V S6-02-6140 Analyzer ELISPOT instrument with BioSpot Professional built-in image analysis (C.T.L).

### Calculation of FRNT_50_

All statistics were performed in GraphPad Prism version 9.4.1. Fit to determine FRNT_50_, and linear regression was performed using custom code in MATLAB v.2019b. Limits of quantification were between 1:25 (most concentrated plasma used) and 1:3200 (most dilute plasma used).

Neutralization data were fit to:

1 $${T}{x}=1/1+(D/{{{\rm{I}}}}{{{{\rm{D}}}}}_{50}).$$

Here, Tx is the number of foci at plasma dilution D normalized to the number of foci in the absence of plasma on the same plate. ID_50_ is the plasma dilution, giving 50% neutralization. FRNT_50_ = 1/ID_50_. Values of FRNT_50_ < 1 are set to 1 (undiluted), the lowest measurable value. We note that FRNT_50_ < 25 or FRNT_50_ > 3200 fell outside of the dilution series used and were extrapolated from the fit.

### Logistic regression to calculate odds ratio

Neutralization status (high vs. low) was modeled as a binary outcome by logistic regression using the glm package in R version 4.3.2. NLR, lymphocyte, CD4 T cell concentrations, and age were dichotomized into a risk group/pathological range (NLR > 6, lymphocytes<1100 cells/µL, CD4 count<350 cells/µL, and age>50), versus normal range. The other predictors (sex, requirement for supplemental oxygen, HIV status) were already categorical. Separate univariate models were fit for each predictor (neutralization (binomial) ~ predictor), followed by a multivariate model (neutralization ~ age + supplemental oxygen + sex + NLR + comorbidities + HIV status + CD4 count + lymphocytes), all using dichotomized values. For each predictor, the regression coefficient from the logistic model was exponentiated to obtain an odds ratio (OR). Ninety-five percent confidence intervals were derived from the likelihood profile of the fitted model and then transformed to the OR scale. A two-sided Wald test *p*-was used to test the null hypothesis that the coefficient equals zero, and *p*-values recorded from the summary.glm R output file.

### Plasma proteomic profiling

Proteomic analysis of the plasma samples was performed by SomaLogic Inc. (Boulder, CO, USA) using the SomaScan v4.0 platform^48^. The SomaScan measures were reported as relative fluorescence units (RFU) in a summary ADAT file. These data were then merged with our metadata. Quantile normalization and log transformation were performed on all RFU-reported data to ensure comparability and normalization across samples.

### Determining differentially expressed proteins

The proteome changes attributable to neutralization capacity, anti-spike protein antibody titer, and disease severity were derived from comparisons between individuals with high versus low neutralization capacity, high versus low anti-spike protein titer, and severe versus non-severe outcomes, respectively. Multiple aptamers of the same protein were averaged. *P*-values were derived using a 2-tailed unpaired *t*-test of log-transformed individual protein levels between comparison groups and corrected to control for the false discovery rate (FDR) using the Storey method^49^. We used this method as opposed to Benjamini-Hochberg because the Storey is a less stringent correction, and we aimed to obtain the maximal number of differentially expressed proteins so as not to underestimate overlaps in differentially regulated proteins between the neutralization and disease severity/spike antibody responses. Proteins with an FDR < 0.05 were considered differentially expressed. To determine proteins associated with numerical neutralization values, log-transformed protein values were regressed against FRNT_50_ using linear regression. *P*-values of the coefficient estimates were adjusted, and significantly associated proteins were determined as those with an FDR < 0.05 using the Storey method. Differentially expressed and significantly associated proteins were then visualized using a volcano plot in the R Project for statistical computing and graphical representation. We also assessed whether the associations between neutralization associated proteins were independent of supplemental oxygen, our indicator of severity. Using the numeric approach, we added the supplemental oxygen variable as a covariate to the protein in each of the linear regressions. No proteins were significantly associated with neutralization in the adjusted numeric analysis.

### Bootstrap to determine frequency of 2 or fewer significant proteins with reduced sample size

To understand whether the low number of significantly differentially expressed proteins for neutralization when participants requiring supplementary oxygen were removed from the analysis were purely because of reduced participant numbers, we assessed the likelihood of observing two or fewer significant proteins by differential protein analysis given the reduced sample size (*n* = 54 with 22 non-severe high neutralizers and 32 non-severe low neutralizers) by bootstrap resampling the high versus low neutralizer comparison. We randomly sampled 22 participants from the high neutralizers and 32 participants from the low neutralizers from the full group of participants (including those with supplemental oxygen) with replacement and conducted differential protein analysis for 1000 iterations. We recorded the number of proteins with an FDR < 0.05 with each analysis, yielding a distribution used to estimate the likelihood of observing two or fewer proteins given the smaller sample sizes of our comparative groups.

### SARS-CoV-2 spike enzyme-linked immunosorbent assay (ELISA) for anti-spike antibody levels

Two μg/mL of spike protein was used to coat 96-well, high-binding plates and incubated overnight at 4 °C. The plates were incubated in a blocking buffer consisting of 5% skimmed milk powder, 0.05% Tween-20, 1×PBS. Plasma samples were diluted to a 1:100 starting dilution in a blocking buffer followed by a series of seven 3-fold serial dilutions. The secondary antibody was diluted to 1:3000 in blocking buffer and added to the plates followed by TMB substrate (Thermofisher Scientific). Upon stopping the reaction with 1 M H_2_SO_4_, absorbance was measured at a 450 nm wavelength. The CR3022 mAb was used as positive control, and Palivizumab was used as negative control. To quantify, the reciprocal of the dilution that gives 50% of maximal binding (EC_50_) was calculated using Prism 10.4.1 software by the built in 4 parameter non-linear regression curve fitting function.

### Gene set enrichment analysis

Gene Set Enrichment Analysis (GSEA) was performed using the Broad Institute GSEA software version 4.3.3^50,51^, the MSigDB Hallmark gene sets (v2023.2), and the UniProt Human Collection chip platform. The GSEA software was downloaded from https://www.gsea-msigdb.org/gsea/downloads.jsp, and GSEA was performed using default parameter settings except for Number of permutations = 10,000; Permutation type = gene_set; 10 ≤ gene set size ≤500.

### Logistic regression prediction model of neutralization outcome

Participants were split into training (60%, *n* = 42) and testing (40%, *n* = 29) sets. To identify a subset of proteins with the most potential predictive power of neutralization outcome, the training data were subjected to differential protein analysis as described above with the addition of fold-change threshold (1.5-fold). This yielded 12 significantly differentially expressed proteins (DEPs) between high and low neutralizers in the training data. The protein data in the training and testing sets were log-transformed and scaled by subtracting the mean and dividing by the standard deviation, with the mean and standard deviation of the training data used for both the training and testing sets. Using bootstrapping (1000 iterations with 100 participant values drawn with replacement per iteration), the neutralization response was iteratively modeled against the 12 DEPs and subjected to forward and backward stepwise regression, selecting the most significant predictors in a regression model by adding (forward) or subtracting (backward) predictors in a stepwise manner, optimizing for Akaike’s information criterion (AIC). With each bootstrap iteration, the proteins selected by stepwise regression were added to a running tally, which served to rank the predictive power of the 12 DEPs upon completion of the bootstrapping. Neutralization response was fitted against the top three ranked proteins, MLN, FAP, and HSPA8, in a binomial logistic regression model, which was trained using the training data. The performance of this multivariate model and the constituent univariate models was assessed using the testing data of ROC curves, and AUC statistics were generated using the R pROC package.

## Results

### Cohort characteristics

We analyzed blood samples from 71 SARS-CoV-2 qPCR positive hospitalized participants (Table 1) infected during the first, ancestral SARS-CoV-2 infection wave in Durban, South Africa (March to October 2020^52,53^), enrolled in a prospective observational longitudinal study. All participants were admitted to hospital for clinical support and/or quarantine. Participants who were profoundly immunocompromised because of advanced HIV disease (CD_4_ T lymphocyte cell counts<50 cells/µL^54^) were excluded from this analysis because of our previous work showing that such severe immunocompromise abrogates the neutralizing antibody response to infection^52,53,55^.

**Table 1:** Participant characteristics by neutralization capacity

	All (*n* = 71)	High Neut. (*n* = 35)	Low Neut. (*n* = 36)	*p*-value*
Age (median, IQR)	42 (34–51)	50 (40–57)	36 (28–46)	<0.001
Sex (male, %)	22 (31%)	15 (43%)	7 (19%)	0.042
Comorbidities (*n*, %)	20 (28%)	17 (49%)	3 (8%)	<0.001
Supp. O_2_ (*n*, %)	17 (24%)	13 (37%)	4 (11%)	0.013
PLWH (*n*, %)	33 (46%)	14 (40%)	19 (53%)	0.34
HIV viremic (*n*, %)	5 (7%)	2 (6%)	3 (8%)	1.0
CD4 nadir (median (IQR) cells/uL)	544 (319–857)	497 (319–700)	594 (389–899)	0.36
Lymph. nadir (median (IQR) cells/uL)	1570 (1230–1980)	1540 (1185–1735)	1650 (1330–2063)	0.19
NLR max. (median, IQR)	2.4 (1.8–5.2)	2.8 (1.7–6.8)	2.3 (1.9-3.3)	0.39
D614G neut. (GMT FRNT_50_)	244	1348	46	<0.001

^*^p-values derived using the Kruskal–Wallace non-parametric test (age, CD4 nadir, lymph. nadir, NLR max., and D614G neut.) or the Fisher’s Exact test (all others).

*Comorbidities* diabetes, hypertension, or both, *Supp. O*_2_ required supplemental oxygen, *PLWH* People living with HIV. Lymph nadir Minimum lymphocyte count during tracking period, *NLR max* Highest neutrophil to lymphocyte ratio during tracking period, *D614G neut* Neutralization of ancestral SARS-CoV-2 with the D614G mutation as determined by the FRNT assay.

**Fig. 1:**
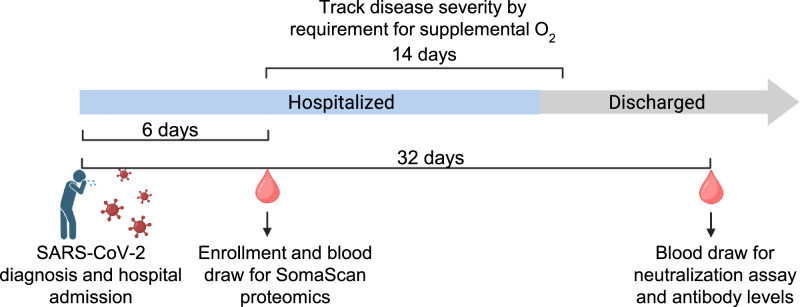
Study design and timeline. Hospitalized study participants (*n* = 71) were enrolled in the first South African SARS-CoV-2 infection wave between March and October 2020. Enrollment occurred as soon as practicable post-hospital admission and was a median of 6 days (IQR 4–8 days) post-diagnosis. Disease severity was assessed by whether a participant required supplemental oxygen at any time during the hospitalization period. A blood draw was performed during the enrollment visit and used for SomaScan proteomic analysis. A second blood draw was performed at 32 days post-diagnosis, and blood plasma was used in a live virus neutralization assay against ancestral SARS-CoV-2 to assess neutralization capacity, as well as for anti-spike antibody levels. Figure created in BioRender.

Study enrollment occurred as soon as practicable after hospital admission, a median of 6 days (IQR 4–8 days) post-SARS-CoV-2 diagnosis by a qPCR test (Fig. 1). A blood sample was taken at the enrollment visit and used for SomaScan proteomic analysis of blood plasma. Participants were tracked by weekly study visits in the first month post enrollment. Hospital discharge usually occurred between the second and third study visits. A second blood sample was taken to measure neutralizing and anti-spike antibody levels at a median of 32 days (IQR 17-35 days) post-diagnosis, usually but not always as part of an outpatient visit (Fig. 1). This timepoint was in the convalescence phase, when the infection elicited antibody response should be fully formed^21^. No participant died before discharge or was admitted to the ICU. Disease severity was determined by whether a participant required supplemental oxygen^27^ at any time during the tracking period from enrollment to first outpatient or, if lost to follow-up, last inpatient visit (Fig. 1, Methods). The duration of the tracking period was a median of 14 days (IQR 7–14 days). Seventeen participants required supplemental oxygen during the tracking period (Table 1), but none required high-flow oxygen or ventilation, indicating that they were not in the top categories of disease severity as defined by the World Health Organization ordinal scale^27^. No supplemental oxygen was administered post-discharge.

Measurement of neutralization was done by a live virus focus reduction neutralization test (FRNT)^56,57^ on the convalescent blood plasma. The sample at convalescence was also used to measure anti-spike antibodies^58^. We determined HIV status, HIV viral load, CD4 T cell concentrations, lymphocyte concentrations, NLR, and whether participants had diabetes or hypertension. Thirty-three (46%) participants were people living with HIV (PLWH, Table 1), reflecting the high HIV prevalence in the province of KwaZulu-Natal, where Durban is located^59^. Median age was 42 years, 22 (31%) were male, and 20 participants (28%) had either hypertension or diabetes, which we combined into one category (comorbidities) to increase statistical power.

### Higher disease severity associates with higher neutralization

**Fig. 2:**
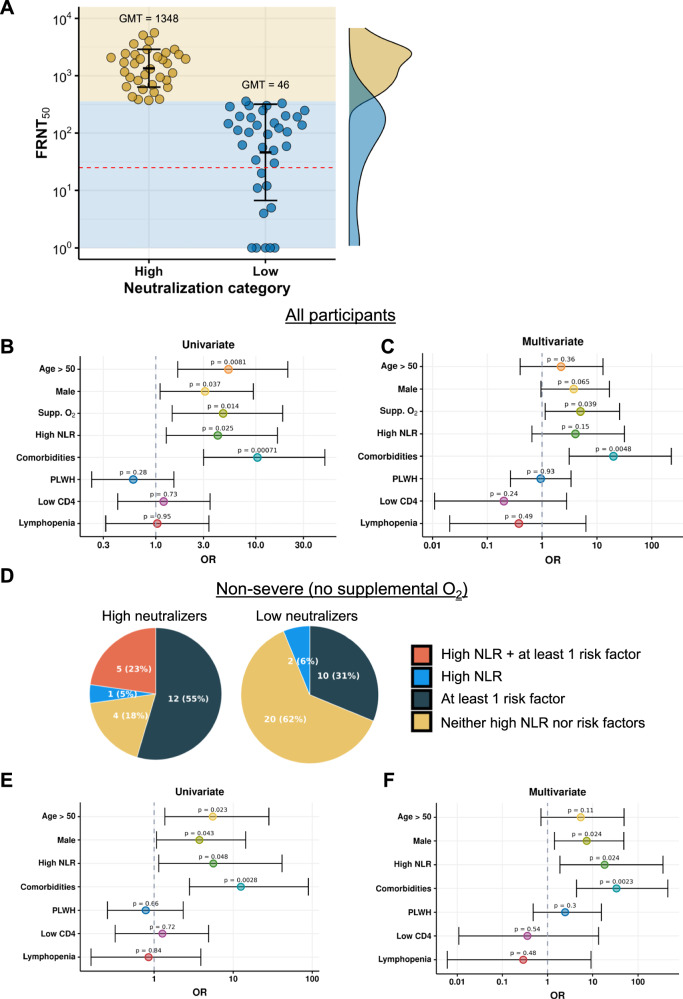
Neutralization capacity associates with disease severity and comorbidities. **A** Participants were divided into high (*n* = 35) and low (*n* = 36) neutralizers based on whether the participant’s plasma showed higher or lower neutralizing capacity relative to the median FRNT_50_ of the full group of participants (*n* = 71). Horizontal dashed red line marks the limit of quantification, which is the inverse of the highest plasma concentration used. The geometric mean titer (GMT) for each group is represented by a horizontal line, and value is shown above the points. Error bars represent the geometric mean titer standard deviation. **B** Univariate and **C** multivariate odds ratios (OR) to be a high neutralizer in the full group of participants (*n* = 71). Each marker shows the point estimate of the OR, horizontal lines show the 95% confidence interval. *P*-values for the odds ratios were derived by a two-sided Wald test. Supp. O_2_: required supplemental oxygen. High NLR: neutrophil to lymphocyte ratio>6. Comorbidities: Hypertension or diabetes, or both. PLWH: People living with HIV. Low CD4: blood CD4 T cell concentration<350 cells/µL. Lymphopenia: blood lymphocytes<1100 cells/µL. **D** Proportion of non-severe (did not require supplemental oxygen, *n* = 54 participants total) high neutralizers (*n* = 22) and low neutralizers (*n* = 32) with either high NLR, at least one risk factor (age, male, comorbidities) for more severe disease, or both. **E** Univariate and **F** multivariate OR to be a high neutralizer in the non-severe group of participants. Markers show the point estimate of the OR, horizontal lines show the 95% confidence interval. with *p*-values determined by a two-sided Wald test.

We present blood plasma neutralization capacity as FRNT_50_, the inverse serum dilution in the FRNT assay required to neutralize 50% of SARS-CoV-2 infectious units. We used the median FRNT_50_ neutralization level (FRNT_50_ = 358) to categorize participants into high and low neutralizers. This gave 36 participants with below median FRNT_50_ and 35 participants with above median FRNT_50_ (Table 1). The geometric mean titer (GMT) FRNT_50_ was 243 for the full group, 46 for the below-median sub-group, and 1346 in the above median sub-group, a 29-fold difference (*p* < 0.001, Fig. 2A and Table 1). We categorized participants in the top half of neutralization values as high neutralizers and bottom half as low neutralizers. The majority of participants who required supplemental oxygen were high neutralizers (13 of 17, *p* = 0.013 by Fisher’s Exact test, Table 1), consistent with the relationship between disease severity and neutralization found in previous studies^13–24^.

To examine other correlates of disease severity in addition to supplemental oxygen, we compared the maximum NLR and minimum (nadir) lymphocyte concentrations in the blood between high and low neutralizers during the tracking period. The median values (Table 1) between the high (NLR = 2.8, lymphocytes = 1540 cells/µL) and low neutralizer groups (NLR = 2.3, lymphocytes = 1650 cells/µL) were not significantly different from each other and not in the range considered to associate with high disease severity^29,35^. In contrast, the high neutralizer group showed a significantly higher fraction of individuals with hypertension, diabetes, or both, and a higher fraction of males. High neutralizers were also older (Table 1). Older age, male sex, and diabetes and hypertension are risk factors for more severe disease^38,60^.

Since CD_4_ T cells are essential to the process of making potent neutralizing antibodies^12^, and CD_4_ T cell concentrations are reduced in unsuppressed or poorly suppressed HIV infection, we compared HIV prevalence, the proportion of HIV viremic individuals, as well as CD_4_ counts in the blood between the high and low neutralizer groups. There was no significant difference in HIV prevalence, and no significant difference in the relatively small number of HIV viremic individuals between the high and low neutralizer groups (Table 1). Median CD_4_ counts (497 cells/µL for high neutralizers versus 594 cells/µL for low neutralizers) were similar and not in the range considered to lead to compromised immune function^61,62^.

To determine the association of each variable with a strong neutralization response, we performed univariate and multivariate regression analysis. We dichotomized the continuous variables (NLR, lymphocyte, CD_4_ T cell concentrations, and age) into a risk group/pathological range (NLR > 6^29^, lymphocytes<1100 cells/µL^35^, CD_4_ count<350 cells/µL^61,62^, and age>50^60^) versus the non-risk/non-pathological range. The number of participants in each category is presented in Table S1. Univariate analysis showed that age over 50, male sex, requirement for supplemental oxygen, NLR in the pathological range, and presence of comorbidities were significantly associated with increased odds of being a high neutralizer (Fig. 2B, odds ratios (OR), confidence intervals, and *p*-values listed in Table S2). In multivariate analysis, the requirement for supplemental oxygen (OR = 5.0, 95% CI 1.1 - 25.9, *p* = 0.04) and comorbidities (OR = 20.1, 95% CI 3.1-228.3, *p* = 0.005) remained significantly associated with high neutralization capacity (Fig. 2C, Table S2). We note the ORs have wide confidence intervals due to low participant numbers and should be interpreted qualitatively because of low numbers in each category.

We were interested to understand what differentiates the non-severe high neutralizers from non-severe, low neutralizers. Fifty-four participants were scored as non-severe because they did not require supplemental oxygen. The non-severe group included 22 high neutralizers and 32 low neutralizers. The GMT FRNT_50_ was 147 for the full group, 39 for the low neutralizers, and 1024 for the high neutralizers, a 26-fold difference (p < 0.001, Table S3). High neutralizers were significantly older (median 48 versus 36 years, Table S3) and had a significantly higher proportion of comorbidities (46% versus 6%, Table S3). While non-significant, there was a higher proportion of males in the high neutralizer group.

NLR, lymphocyte, CD4 T cell concentrations, and age were dichotomized for the non-severe participants in the same way as for the full group of participants. The number of non-severe participants was small, so in order to observe trends we grouped some of the parameters and compared the proportion of participants in non-severe high versus low neutralizers with: (1) pathologically high NLR and at least one risk factor for more severe disease (age, male sex, and comorbidities); (2) high NLR only; (3) risk factor only; (4) normal NLR and no risk factors. We found that in the low neutralizer group, 60% had a normal NLR and no risk factors. In contrast, less than 20% had a normal NLR and no risk factors in the high neutralizer group (Fig. 2D).

We performed univariate and multivariate analysis on the non-severe low versus high neutralizers. We note the ORs have wide confidence intervals and should be interpreted qualitatively. In univariate analysis, age, male sex, high NLR, and comorbidities were significantly associated with increased odds of being a non-severe high-neutralizer (Fig. 2E OR, confidence intervals, and *p*-values listed in Table S4). In multivariate analysis, male sex, high NLR, and comorbidities remained associated with increased odds of being a high-neutralizer (Fig. 2F and Table S4). This may indicate that there are still gradations in Covid-19 severity in the lower severity participants who did not require supplemental oxygen. The high neutralizers who did not require supplemental oxygen still likely had higher disease severity relative to low neutralizers who did not require supplemental oxygen.

### Host proteins associated with higher neutralization at convalescence

**Fig. 3:**
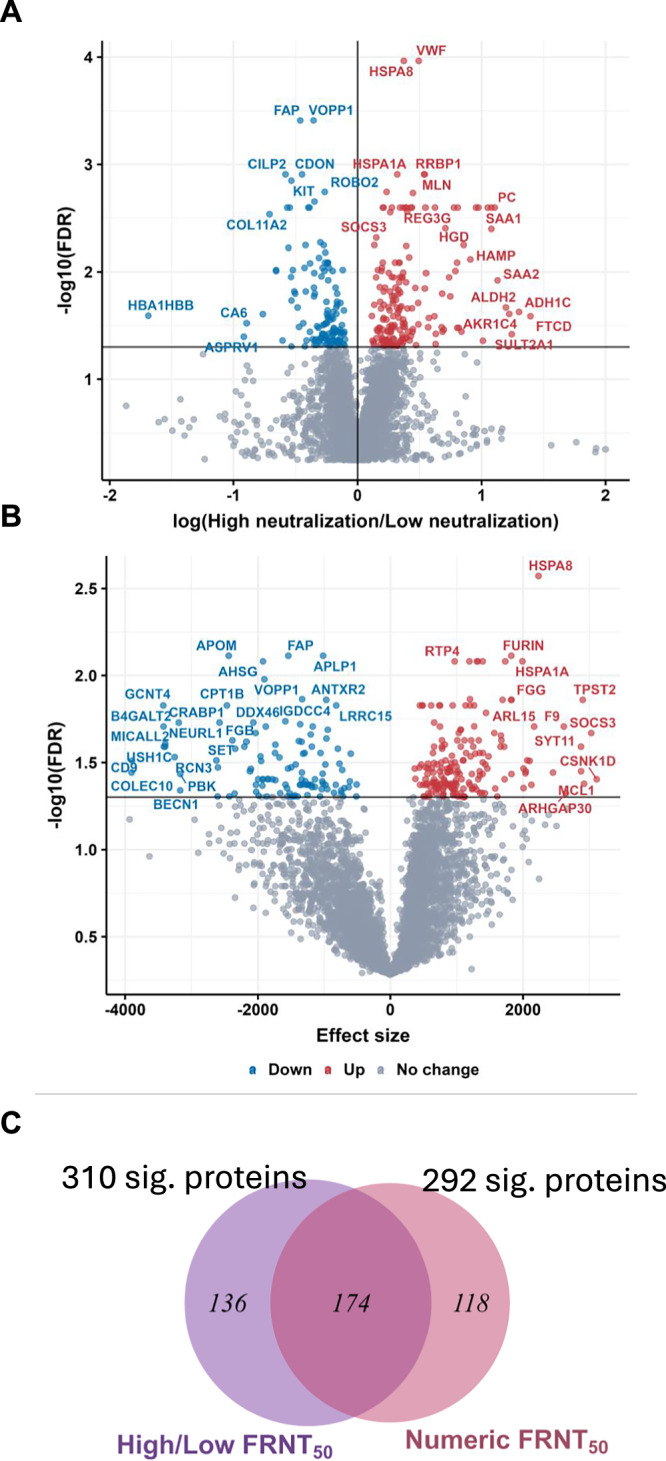
Differentially expressed proteins in high versus low neutralizers. **A** Volcano plot of false discovery rate (FDR) versus fold-change for each protein in participants who are high neutralizers (*n* = 35) compared to low neutralizers (*n* = 36) using the categorical approach. *P*-values were derived using a 2-tailed unpaired t-test of individual log-transformed protein values between comparison groups. *P*-values were adjusted using the Storey method to obtain the FDR. **B** Volcano plot of false discovery rate (FDR) versus effect size (as R^2^) for each protein in a regression of FRNT_50_ against protein levels for the full group of participants (*n* = 71). *P*-values of the coefficient estimates were adjusted using the Storey method to obtain the FDR. Proteins with higher expression in high neutralizers are in red, and those with lower expression are in blue. **C** Venn diagram showing the number of significantly associated proteins (FDR < 0.05) that overlap for the categorical (high versus low neutralizers) and regression (numeric) approaches.

We used SomaScan proteomics^63^ to determine the levels of ~5000 proteins in participant plasma from the blood sample collected at enrollment, which was a median of 6 days after the time of diagnosis. We used two analysis approaches to detect significant associations between protein levels at enrollment with neutralization levels at convalescence to increase confidence that the results are independent of the specific analysis approach used. In the first approach, we used participants dichotomized into high and low neutralizer groups (Fig. 2A) to find differentially expressed proteins (DEPs) between the groups. In the second approach, we used linear regression to determine significant associations between protein levels and neutralization values. For both approaches, the *p*-value was adjusted using the false discovery rate (FDR q-value)^64–66^. The approaches differ in what they measure: the first approach quantifies fold-change in protein level between groups, while the second approach quantifies correlation. To make the approaches comparable, we defined significant DEPs as having an FDR < 0.05 and did not threshold on fold-change or effect size in this analysis. We obtained 310 significant proteins using the categorical (high/low neutralizers) approach (Fig. 3A) and 292 significant proteins using the numerical (regression) approach (Fig. 3B). The total number of unique DEPs detected by the combination of both analysis approaches was 428. The overlap obtained between the two approaches was 174 proteins (41% of the total unique proteins, Fig. 3C).

The top DEP by significance (Table 2) using the categorical approach was HSPA8, with higher levels in high neutralizers. This protein is a chaperone from the HSP70 family of constitutively expressed heat shock proteins and interacts with proteins from multiple viruses, including the SARS-CoV-2 spike^67^, the influenza M1^68^, and the papillomavirus L2^69^ proteins. The second most significant DEP with higher levels in high neutralizers was von Willebrand factor (VWF), involved in orchestrating the coagulation response^67^.

**Table 2:** Top ten significantly differentially expressed proteins by condition

Neut. categorical		Neut. numeric		Anti-spike Ab		Severity	
**Protein**	FDR*	**Protein**	FDR**	**Protein**	FDR*	**Protein**	FDR*
HSPA8	0.00011	HSPA8	0.0000011	VOPP1	0.00021	CXCL13	0.0045
VWF	0.00011	APLP1	0.000014	PLAT	0.00023	FURIN	0.0045
FAP	0.00039	APOM	0.000015	VWF	0.00023	CAPN2	0.0045
VOPP1	0.00039	FAP	0.000016	NADK	0.00035	RHOB	0.014
CDON	0.0012	FURIN	0.000014	CILP2	0.00052	DNASE1L2	0.014
CILP2	0.0012	AHSG	0.000031	CR2	0.00057	ARFGAP1	0.014
HSPA1AA	0.0012	DNASE1L2	0.000032	ADAMTSL2	0.0019	CD79A	0.014
MLN	0.0012	HSPA1A	0.000040	ANTXR2	0.0019	CSMD1	0.014
RRBP1	0.0012	RANBP3	0.000043	CDH5	0.0019	APOM	0.017

*Neut* Neutralization, *FDR* False discovery rate.

^a^*p*-values were derived using a 2-tailed unpaired *t*-test of individual log-transformed protein values between comparison groups and corrected to control for the false discovery rate using the Storey method.

**Log-transformed individual protein values were regressed against FRNT_50_ using linear regression. *p*-values of the coefficient estimates were adjusted using the Storey method.

Using the regression approach, we found that HSPA8 was also the top hit by significance and positively correlated with neutralization, with an FDR that was about an order of magnitude more significant than the next most significantly regulated protein (Table 2). Furin and amyloid beta precursor like protein 1 (APLP1) were the next most significant proteins, with Furin upregulated and APLP1 downregulated. Furin is a protease that cleaves the S1/S2 polybasic site and therefore facilitates SARS-CoV-2 fusion and cellular infection^70^, while APLP1 is required for glucose homeostasis^71^, and its downregulation is associated with neurological symptoms of Covid-19^72^. A third protein, which was downregulated with similar significance, was apolipoprotein M (ApoM), involved in lipid function, which may have a protective role in infections^73^. In addition to HSPA8, Fibroblast Activation Protein (FAP), involved in the tissue fibrosis response^74^, came up as significantly upregulated using both analysis approaches, as were the heat shock protein family chaperone HSPA1A^75^ and ribosome-binding protein 1 (RRBP1), involved in the ER stress response^76^.

### Host proteins associated with anti-spike antibody levels

**Fig. 4:**
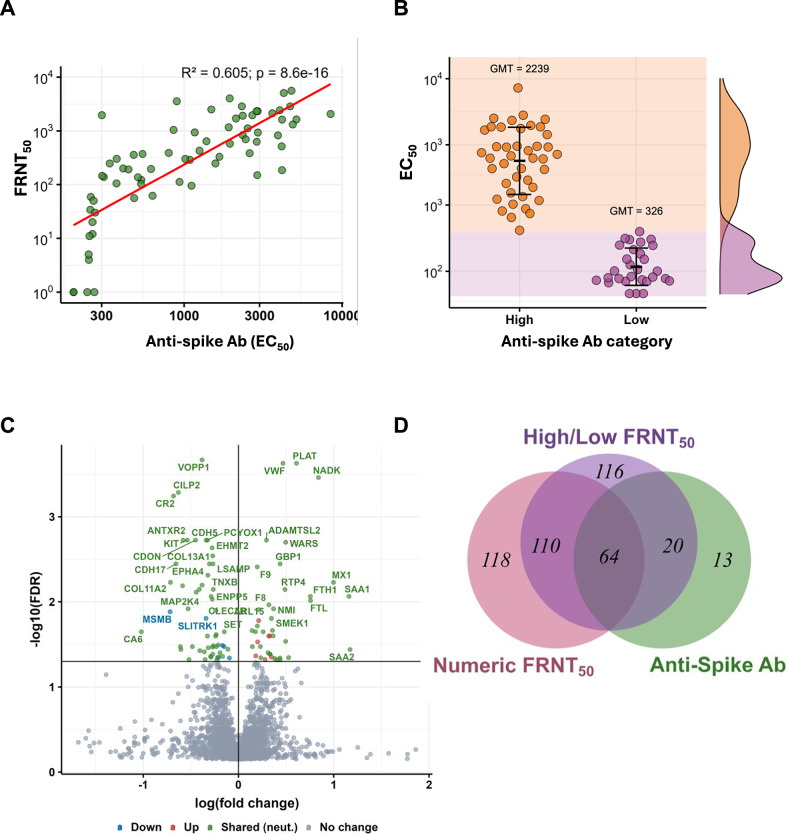
Differentially expressed proteins in participants with high versus low anti-spike antibody levels. **A** Correlation between neutralization as FRNT_50_ and anti-spike antibody levels as EC_50_ (the reciprocal of the dilution that gives 50% of maximal binding). The correlation coefficient (*R*^2^) with the associated *p*-value is shown above the graph. **B** The 60th percentile of EC_50_ was used to separate participants with high (top 60%, *n* = 42) versus low (bottom 40%, *n* = 29) anti-spike antibody levels. The geometric mean titer (GMT) for each group is represented by a horizontal line and shown above the points. Error bars represent the geometric mean titer standard deviation. **C** Volcano plot of false discovery rate (FDR) versus fold-change for each protein. *P*-values were derived using a 2-tailed unpaired t-test of individual log-transformed protein values between participants with high versus low anti-spike antibody levels. FDR correction by the Storey method. Proteins marked in green are also significantly associated with the neutralizing antibody response. **D** Venn diagram showing overlap of significant DEPs associated with high anti-spike antibody levels (green) with DEPs associated with high neutralizers as determined by the categorical (purple) and regression (red) approaches.

In addition to neutralization, we measured anti-spike antibody levels in the same convalescent participant samples. We obtained a strong correlation between FRNT_50_ and anti-spike antibody levels across the participants tested (*R*^2^ = 0.6, p < 0.001, Fig. 4A). For subsequent analysis, we categorized participants into those with high versus low antibody levels. Differences in expression were lower, and to extract the highest number of DEPs, we assessed DEPs at varying thresholds of anti-spike antibody expression to separate high versus low antibody groups. The 60th percentile gave the most DEPs. The difference between the high and low antibody group was 6.9-fold (Fig. 4B), considerably lower than the 29-fold difference we obtained in FRNT_50_ for high versus low neutralizers. Probably because of the lower differences in anti-spike antibody levels between high and low participants, only one protein was significantly associated when using linear regression analysis, and we did not use this approach further.

Using the categorical approach, we obtained 97 significant DEPs (Fig. 4C). The 3 top hits by significance were Vesicular, Overexpressed in Cancer, Prosurvival Protein 1 (VOPP1, also called ECop), involved in the upregulation of the NF-κB pathway, Plasminogen Activator, Tissue Type (PLAT), also called tPA, which mediates the clot breakdown response^77^, and FAP, which is also one of the most significantly regulated proteins in high versus low neutralizers (Table 2). More generally, 84 (87%) of the DEPs in the anti-spike antibody response were common with the DEPs found in the neutralization response, as determined either in the categorical or numerical approach (Fig. 4D, proteins marked in green in Fig. 4C).

### Neutralization associated proteins are linked to disease severity

**Fig. 5:**
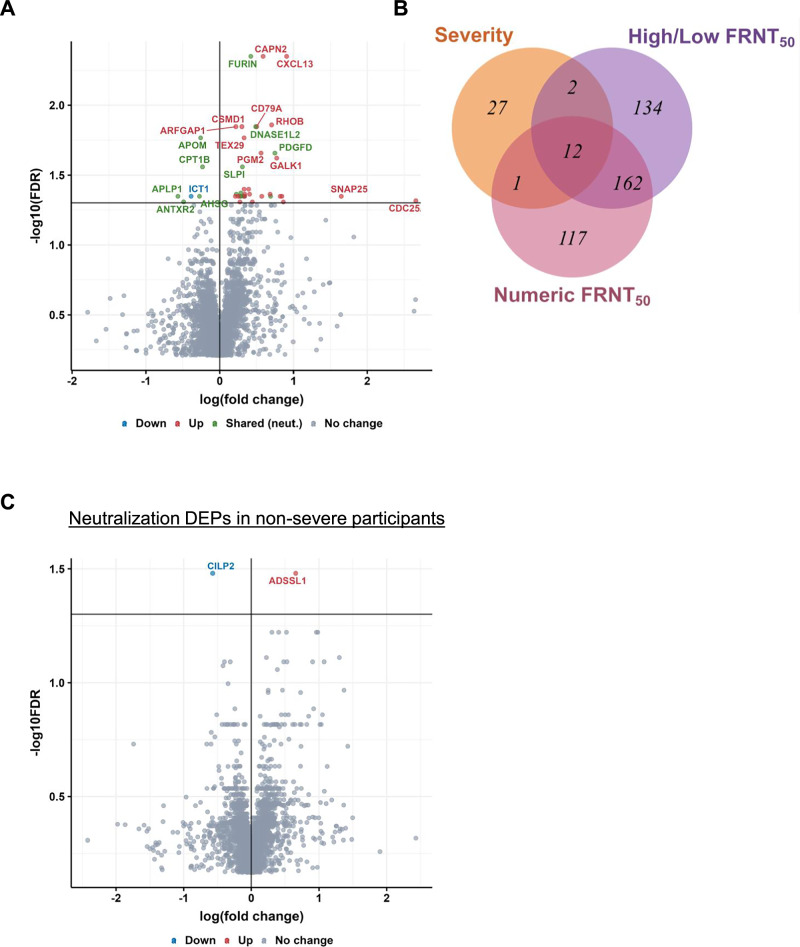
Differentially expressed proteins in participants with higher versus lower disease severity. **A** Volcano plot of false discovery rate (FDR) versus fold-change for each protein in participants with higher versus lower disease severity, where disease severity was determined by the requirement for supplemental oxygen. Proteins marked in green are also significantly associated with the neutralizing antibody response. **B** Venn diagram showing overlap of significant DEPs associated with higher disease severity (orange) and high neutralizers as determined by the categorical (purple) and regression (red) approaches. **C** Volcano plot of false discovery rate (FDR) versus fold-change in high neutralizers versus low neutralizers using the categorical approach when participants who required supplemental oxygen were excluded.

Disease severity, being inherently categorical, was analyzed by comparing protein expression in the group of participants who required supplemental oxygen at any point before discharge (*n* = 17) versus those who did not (*n* = 54). There were 42 significant DEPs (Fig. 5A), a low number relative to other studies^41–44^. This is explainable by the lack of critically ill participants and a low number of participants requiring supplemental oxygen. The top hit by significance (Table 2) was the chemokine CXCL13, a biomarker for increased disease severity in Covid-19^41,78^. The next most significant hits were the protease Furin (in common with the neutralizing response), which promotes SARS-CoV-2 cellular infection by cleaving the S1/S2 polybasic site and facilitating viral fusion^70^, and Calpain-2 (CAPN2), involved in positively regulating the cell surface levels of the ACE2 receptor and hence also expected to promote SARS-CoV-2 infection^79^. In addition to Furin, DNAse1L2 was one of the top hits by significance associated with both disease severity and neutralization (Table 2). DNAse1L2 has been shown to be transcriptionally activated by inflammatory cytokines resulting from infection^80^.

Unlike the strong overlap between neutralization and anti-spike antibody responses (87%), overlap between disease severity and neutralization associated proteins was more modest: 15 of 42 DEPs (36%) in the disease severity response were found to overlap with the neutralization response (Fig 5B, proteins marked in green in Fig. 5A). To further investigate the relationship between disease severity and neutralization associated proteins, we attempted to correct *p*-values of neutralization associated proteins by disease severity using a multivariate model (Methods). This correction resulted in no significant DEPs for high versus low neutralizers. We next tried another approach: we excluded the 17 participants who required supplemental oxygen and compared high versus low neutralizers using the categorical approach on the remaining 22 non-severe high neutralizers and 32 non-severe low neutralizers. We obtained only 2 significant DEPs, with borderline significance (Fig. 5C). To examine whether this was due to low numbers of participants after exclusion, we performed a bootstrap simulation where we picked at random 22 high neutralizers from the full high-neutralizer group (including those requiring supplemental oxygen), and 32 participants from the full low neutralizer group (Methods). We observed that all 1000 iterations gave more than 2 significant proteins (*p* < 0.001, Fig. S1). Our interpretation of the bootstrap result is that the loss of DEPs between high and low neutralizers when participants requiring supplemental oxygen were excluded was not explained by low participant numbers in this subset. Rather, the participants requiring supplemental oxygen account for the majority of the differences in terms of protein expression between high and low neutralizers.

### Differentially regulated pathways between high and low neutralizers

**Fig. 6:**
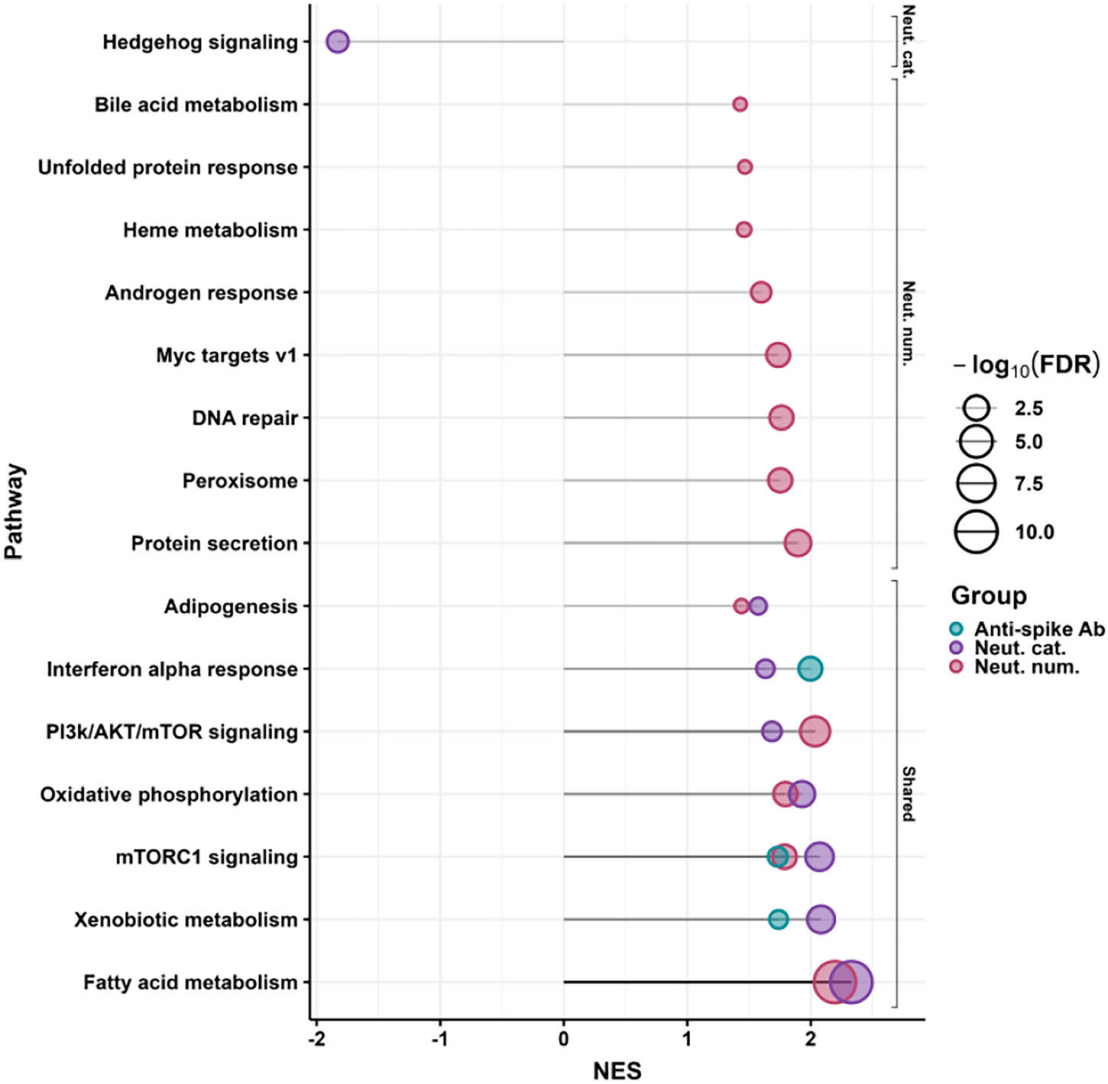
Significantly regulated pathways associated with high neutralization and anti-spike antibody levels. Gene Set Enrichment Analysis (GSEA) results for significantly up- or downregulated pathways associated with the neutralization response based on associated proteins derived by the categorical (purple) or numerical (red) approaches, or significantly upregulated pathways in participants with high versus low spike antibody levels (green). The *x*-axis represents the Normalized Enrichment Score (NES). Each circle represents a pathway, with the size of the circle corresponding to the -log_10_(FDR) value, with larger circles indicating higher significance.

We used Gene Set Enrichment Analysis (GSEA)^50^ to determine the differentially regulated pathways between high and low neutralizers and between those with high versus low anti-spike antibody levels (Fig. 6). We used the Molecular Signatures Database (MSigDB) Hallmark gene set^81^ and a significance threshold of FDR < 0.1 to determine significantly enriched pathways. We found 7 upregulated pathways and 1 downregulated pathway in high versus low neutralizers by the categorical approach, and 13 upregulated pathways using the numerical/regression approach. We found 3 significantly upregulated pathways for anti-spike antibodies, all of them in common with the neutralization categorical approach. We also found 5 upregulated pathways in common between the neutralization categorical approach and the neutralization regression approach.

Pathways that were detected by more than one analysis approach increase our confidence in their involvement in the humeral response to SARS-CoV-2, and we focus our description on these. Pathways in common between the categorical and numerical analysis approaches in neutralization were adipogenesis, PI3k/Akt/mTOR signaling, oxidative phosphorylation (OXPHOS), mTORC1 signaling, and fatty acid metabolism. The latter was the most significantly upregulated pathway. The fatty acid metabolism pathway is closely associated with SARS-CoV-2 replication, with inhibition of this pathway resulting in suppression of viral replication^82–84^. Increased OXPHOS may be involved in the response to infection. For example, activated B cells require increased OXPHOS^85^. Adipogenesis is associated with obesity, a known risk factor for severe Covid-19^86–89^.

The pathways in common between high versus low neutralizers and participants with high versus low anti-spike antibody levels were the interferon-α (IFN-α) response, mTORC1 signaling, and xenobiotic metabolism. The IFN-α response is a key part of the innate immune response to viral infections and involves IFN-α triggering antiviral and pro-inflammatory effects^90^. The mTORC1 pathway is a metabolic regulator of cell growth^91^, with mTORC1 inhibitors shown to reduce SARS-CoV-2 replication^92^. Finally, upregulation of xenobiotic metabolism may indicate the presence of pharmacological interventions^93^, possibly associated with more severe disease.

### Protein signatures for a high infection elicited neutralizing antibody response

**Fig. 7:**
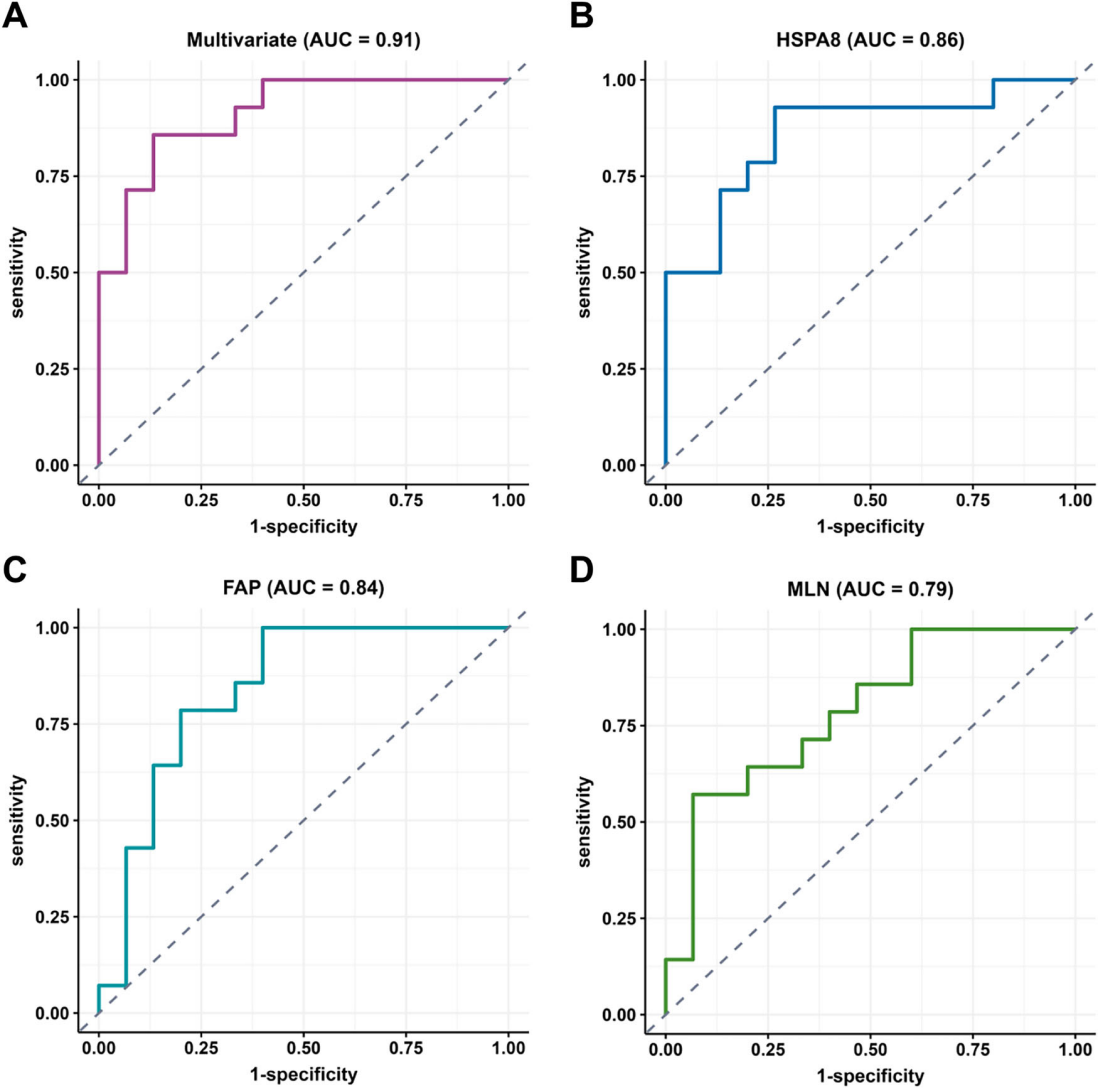
Predictive models classifying participants into high versus low neutralization groups. Shown are ROC curves for (**A**) multivariate (HSPA8 + FAP + MLN), **B** HSPA8, **C** FAP, and **D** MLN. Areas Under the Curve (AUC) were 0.91 (multivariate), 0.86 (HSP8A), 0.84 (FAP), and 0.79 (MLN). Dashed diagonal line represents the performance of a random classifier with AUC = 0.50.

We investigated whether we could predict strong neutralizing antibody responses based on the DEPs significantly associated with neutralization level. The categorical approach was used to separate participants into high and low neutralizers by median FRNT_50_. Participants were split into training (60%, *n* = 42) and test (40%, *n* = 29) groups. Significantly regulated proteins were determined by FDR < 0.05, and to reduce the number of hits, a 1.5-fold threshold in protein level between high and low neutralizers. This resulted in 12 significantly regulated proteins in the neutralization response in the training set. A repeated stepwise regression using bootstrapping was performed to rank the predictive power of each of the 12 proteins in the test set by iteratively subtracting or adding each of the proteins from/to the model. Stepwise regression was performed until the Akaike information criterion (AIC), a measure of the trade-off between goodness-of-fit and model complexity, was optimized. The top three predictive proteins in order of significance were HSPA8, MLN, and FAP. They were combined in a multivariate logistic regression model (Fig. 7A) and analyzed singly in univariate regression (Fig. 7B–D).

The combination of HSPA8, MLN, and FAP resulted in good discrimination between high neutralizers versus low neutralizers, showing an area under the curve (AUC) of 0.91 (Fig. 7A). Using HSPA8 alone, the model could reasonably distinguish between low and high neutralization outcomes in the test group (AUC = 0.86, Fig. 7B). The predictive power of FAP (AUC = 0.84) and MLN (AUC = 0.79) was slightly lower (Fig. 7C, D).

## Discussion

We analyzed protein and neutralizing antibody levels in a cohort of 71 South African participants infected with SARS-CoV-2 during the first ancestral SARS-CoV-2 infection wave. We found host proteins associated with the development of higher neutralization capacity, higher anti-spike antibody levels, and higher disease severity. We also found that higher disease severity and comorbidities were significantly associated with higher neutralization capacity, confirming previous results^13,15,22^. This seemed to be also true in participants with relatively low severity who did not require supplemental oxygen, as there was evidence for gradations of disease severity based on high NLR and the risk factors of age, male sex, and presence of comorbidities, which were associated with higher neutralization. Interestingly, neither HIV status, nor lymphopenia, nor a CD_4_ count<350 cells/µL had significant effects on neutralization. These observations likely indicate that participants who were PLWH were immunocompetent to mount a neutralizing antibody response against SARS-CoV-2^52,53,55^.

Unlike the anti-spike antibody response, where 87% of DEPs associated with higher spike antibody levels were also associated with higher neutralization, only 36% of DEPs associated with higher disease severity were also associated with higher neutralization. This overlap seems smaller than expected if the two processes are highly related. However, excluding participants with higher disease severity who required supplemental oxygen eliminated nearly all significant neutralization associated DEPs. We found that a reduced sample size does not account for this effect. Combined with the evidence for higher gradations of disease severity in high neutralizers relative to low neutralizers who did not require supplemental oxygen, this may indicate that Covid-19 severity is tightly associated with the level of neutralization elicited by SARS-CoV-2 infection. However, given that most high neutralizers (22 out of 35) did not require supplemental oxygen, ARDS is not required for a high neutralizing antibody response. Possibly, both higher disease severity and a potent neutralizing response result from a common factor such as higher levels of SARS-CoV-2 replication.

Interestingly, some of the upregulated enriched pathways we observed in high versus low neutralizers were generally linked with SARS-CoV-2 replication. These included PI3k/Akt/mTOR signaling, which is a mediator of cell cycle progression and cell survival^94^, with activation increasing viral replication^95–97^; OXPHOS, the process by which mitochondria generate ATP, where SARS-CoV-2 infection has been reported to promote OXPHOS and increase ATP production^98^; the mTORC1 pathway, a metabolic regulator of cell growth^91^, with mTORC1 inhibitors shown to reduce SARS-CoV-2 replication^92^; and fatty acid metabolism, essential for the replication of enveloped viruses^83,99,100^. These results were obtained in our cohort of SARS-CoV-2 infected people in South Africa, and given the lack of similar studies, the generalizability to other populations is yet to be determined.

The protein that was most significantly associated with the neutralizing antibody response was HSPA8, an Hsp70 family member. It was also the best single protein predictor of whether an infection will elicit high levels of neutralizing antibodies. HSPA8 has an important role in antigen presentation on MHC class II molecules^45,46^, a necessary step in the CD4 helper T cell - B cell interactions, which are responsible for the production of potent neutralizing antibodies^101,102^. Furthermore, Hsp70 family members have been shown to mediate cross-presentation of extracellular antigens to antigen presenting cells^47^. We note that we performed proteomics on blood plasma. Proteins may be present in this extracellular compartment because of processes such as necroptosis, which leads to rapid membrane permeabilization and the passive release of cellular contents after cell death^103^. Therefore, one mechanism for HSPA8 promoting SARS-CoV-2 neutralization may be that, due to more infection and consequent cell death, HSPA8 can cross-present more extracellular viral spike proteins to antigen presenting cells. These cells take up the HSPA8-spike complex with scavenger receptors^47^ and activate SARS-CoV-2 spike-specific CD4 T cells, leading to increased T cell help to spike-specific B cells.

The limitations of the study include the overall small cohort size, uncertainty in the exact time of infection, unknown viral titer, and the low number of participants with higher disease severity, as none of the participants died, was ventilated, administered high flow oxygen, or admitted to the ICU. Participants with moderate disease, as scored by the requirement for supplemental oxygen, constituted less than a quarter of the cohort. Therefore, unlike other studies^41–44^, we only had a small number of proteins (42 in total) associated with disease severity. Among the disease severity proteins we detected, the most significantly regulated DEPs were CXCL13, Furin, and CAPN2. The cytokine CXCL13 is a biomarker for increased disease severity in Covid-19 and one of two proteins that constituted a predictive proteomic signature for severe disease in one study^41,78^. Furin was found to be associated with disease severity in several studies^43,44^. CAPN2, involved in positively regulating the cell surface levels of the SARS-CoV-2 ACE2 receptor^79^, would be expected to increase SARS-CoV-2 infection but was not found to be associated with disease severity in other proteomic studies. Other proteins central to the disease severity response, such as IL6^41–44,104^, were not found in our analysis. Therefore, the degree of overlap between proteins associated with disease severity and neutralization may be more extensive than estimated here.

There was a strong overlap in the proteins associated with higher levels of spike binding antibodies and those associated with neutralization. The lower number of significant DEPs (97 in total) associated with the anti-spike antibody response may be due to the lower fold change between participants with high versus low spike antibody levels (6.9-fold) relative to the high versus low neutralizers (29-fold). The strong overlap may indicate that some of the proteins in the neutralization response increase the quantity of antibodies as opposed to antibody potency. However, the proteins that best predict the level of neutralization, such as HSPA8, do not appear as top hits associated with the anti-spike response. In addition, the larger number of proteins associated with neutralization, and the much larger fold difference in neutralization levels between high and low neutralizers, may indicate that some of the neutralization associated proteins are involved in modulating neutralizing potency.

In summary, we show that host proteins and pathways involved early in the response to SARS-CoV-2 infection significantly associate with neutralizing antibody levels elicited by the infection. These results may be a step toward understanding neutralizing antibody response heterogeneity between infected individuals and in deciphering how a robust neutralizing antibody response to infection can be induced.

## Supplementary information

Supplemental Information

Description of Additional Supplementary Files

Supplementary Data 1

## Data Availability

The full SomaScan® proteomic dataset supporting this study is publicly available in Zenodo under the 10.5281/zenodo.17551035^105^. The repository contains the raw.adat file from the SomaLogic PharmaServices v4 platform, processed data tables used for downstream analyses, and metadata describing each sample and variable. The ancestral SARS-CoV-2 isolate used for neutralization assays has been deposited in GISAID (EPI_ISL_602626.1) and GenBank (OP090658), and the virus is available upon reasonable request. Data used to generate all figures in this work are available in Excel format as Supplementary Data 1 associated with this manuscript. In addition, data underlying figures along with metadata describing each sample and variable, are available in the GitHub repository on https://github.com/Afrah-Khairallah/somascan-host-proteins-sarscov2 and archived in Zenodo (10.5281/zenodo.17609054)^106^, with figure-specific datasets detailed in the README file on GitHub.

## Code availability

All scripts for data processing and figure generation are available on https://github.com/Afrah-Khairallah/somascan-host-proteins-sarscov2. The repository is archived on Zenodo (10.5281/zenodo.17609054)^106^. The repository contains all scripts used for data preprocessing, statistical analysis, and gene set enrichment analyses.
