# Supplementary material for: Host proteins associated with strong neutralizing SARS-CoV-2 antibody responses in a South African cohort
Source: Commun Med (Lond). 2026 Feb 13;6:203. doi: 10.1038/s43856-026-01427-7 (PMC13066479; doi:10.1038/s43856-026-01427-7)
Supplement: Supplementary file 1 — Supplemental Information [file 43856_2026_1427_MOESM1_ESM.pdf]

Supplementary Information

Khairallah et al.

Contents

Supplementary Tables 2
Table S1: Numbers of high and low neutralizers in each risk category 2
Table S2: Odds ratios full participant group (dependent variable: high vs. low neutralization) 2
Table S3: Participant characteristics of non-severe participants grouped by neutralization capacity 3
Table S4: Odds ratios non-severe group (dependent variable: high vs. low neutralization) 3
Supplementary Figures 4
Figure S1: Frequency of 2 or fewer significant proteins with reduced sample size using full participant group 4
Figure S2: Significantly differentially expressed proteins in the training set 5

Supplementary Tables

Table S1: Numbers of high and low neutralizers in each risk category

	High neutralizers (n=35)	Low neutralizers (n=36)
**Age > 50**	14	4
**Male**	15	7
**Supp. O_2**	13	4
**High NLR**	12	4
**Comorbidities**	17	3
**PLWH**	14	19
**Low CD4**	10	9
**Lymphopenia**	7	7

Supp. O_2: required supplemental oxygen. High NLR: neutrophil to lymphocyte ratio>6. Comorbidities: diabetes, hypertension, or both. PLWH: People living with HIV. Low CD_4: CD4 count <350 cells/μL. Lymphopenia: Lymphocyte count<1100 cells/μL.

Table S2: Odds ratios full participant group (dependent variable: high vs. low neutralization)

	**Univariate**		**Multivariate**	
	OR (95% CI)	p-value	OR (95% CI)	p-value
**Age > 50**	5.33 (1.66 - 20.87)	0.0081	2.21 (0.40 - 12.90)	0.36
**Male**	3.10 (1.10 - 9.45)	0.04	3.78 (0.96 - 16.88)	0.07
**Supp. O_2**	4.73 (1.46 - 18.56)	0.01	5.02 (1.14 - 25.93)	0.04
**High NLR**	4.17 (1.27 - 16.46)	0.03	4.06 (0.65 - 31.91)	0.15
**Comorbidities**	10.39 (3.00 - 48.93)	>0.001	20.05 (3.12 - 228.34)	0.0048
**PLWH**	0.60 (0.23 - 1.52)	0.28	0.95 (0.27 - 3.37)	0.93
**Low CD4**	1.20 (0.42 - 3.49)	0.73	0.20 (0.01 - 2.80)	0.24
**Lymphopenia**	1.04 (0.32 - 3.40)	0.95	0.38 (0.02 - 6.33)	0.49

Supp. O_2: required supplemental oxygen. High NLR: neutrophil to lymphocyte ratio>6. Comorbidities: diabetes, hypertension, or both. PLWH: People living with HIV. Low CD_4: CD4 count <350 cells/μL. Lymphopenia: Lymphocyte count<1100 cells/μL. OR: odds ratio. CI: confidence interval. P-values were derived by a two-sided Wald test.

Table S3: Participant characteristics of non-severe participants grouped by neutralization capacity

	All (n=54)	High Neut. (n=22)	Low Neut. (n=32)	p-value*
**Age (median, IQR)**	42 (32 - 50)	48 (42 - 56)	36 (27 - 46)	0.0011
**Sex (male, %)**	14 (26%)	9 (41%)	5 (16%)	0.058
**Diabetes or hypertension (n, %)**	12 (22%)	10 (46%)	2 (6%)	0.002
**PLWH (n, %)**	24 (44%)	9 (41%)	15 (47%)	0.78
**HIV viremic (n, %)**	3 (5.6%)	3 (9.4%)	0 (0.0%)	0.49
**CD4 nadir (median (IQR) cells/uL)**	587 (464–888)	504 (382–724)	615 (469–930)	0.46
**Lymph. nadir (median (IQR) cells/uL)**	1625 (1310–2053)	1585 (1252–1982)	1650 (1340–2062)	0.62
**NLR max (median, IQR)**	2.3 (1.8–3.8)	2.4 (1.5–5.8)	2.3 (2.0–2.9)	0.92
**D614G neut. (GMT $FRNT_{50}$)**	147	1024	39	<0.001

*p-values derived using the Kruskal-Wallace non-parametric test (age, CD4 nadir, lymph. nadir, NLR max., and D614G neut.) or the Fisher's Exact test (all others). Comorbidities: diabetes, hypertension, or both. Supp. O2: required supplemental oxygen. PLWH: People living with HIV. Lymph. nadir: Minimum lymphocyte count during tracking period. NLR max.: Highest neutrophil to lymphocyte ratio during tracking period. D614G neut.: Neutralization of ancestral SARS-CoV-2 with the D614G mutation as determined by the FRNT assay. P-values were derived by the non-parametric Kruskal-Wallace test.

Table S4: Odds ratios non-severe group (dependent variable: high vs. low neutralization)

	**Univariate**		**Multivariate**	
	OR (95% CI)	p-value	OR (95% CI)	p-value
**Age > 50**	5.52 (1.37–28.31)	0.02	5.42 (0.71–49.43)	0.11
**Male**	3.74 (1.07–14.38)	0.04	7.37 (1.41–48.86)	0.02
**High NLR**	5.63 (1.15–41.53)	0.05	18.22 (1.87–358.93)	0.02
**Comorbidities**	12.50 (2.79–89.68)	0. 003	33.46 (4.37–459.68)	0.002
**PLWH**	0.78 (0.26–2.34)	0.66	2.44 (0.48–15.43)	0.30
**Low CD4**	1.27 (0.32–4.90)	0.72	0.36 (0.01–13.40)	0.54
**Lymphopenia**	0.85 (0.16–3.91)	0.84	0.29 (0.01–9.17)	0.48

Supp. O_2: required supplemental oxygen. High NLR: neutrophil to lymphocyte ratio>6. Comorbidities: diabetes, hypertension, or both. PLWH: People living with HIV. Low CD_4: CD4 count <350 cells/μL. Lymphopenia: Lymphocyte count<1100 cells/μL. OR: odds ratio. CI: confidence interval. P-values were derived by a two-sided Wald test.

Supplementary Figures

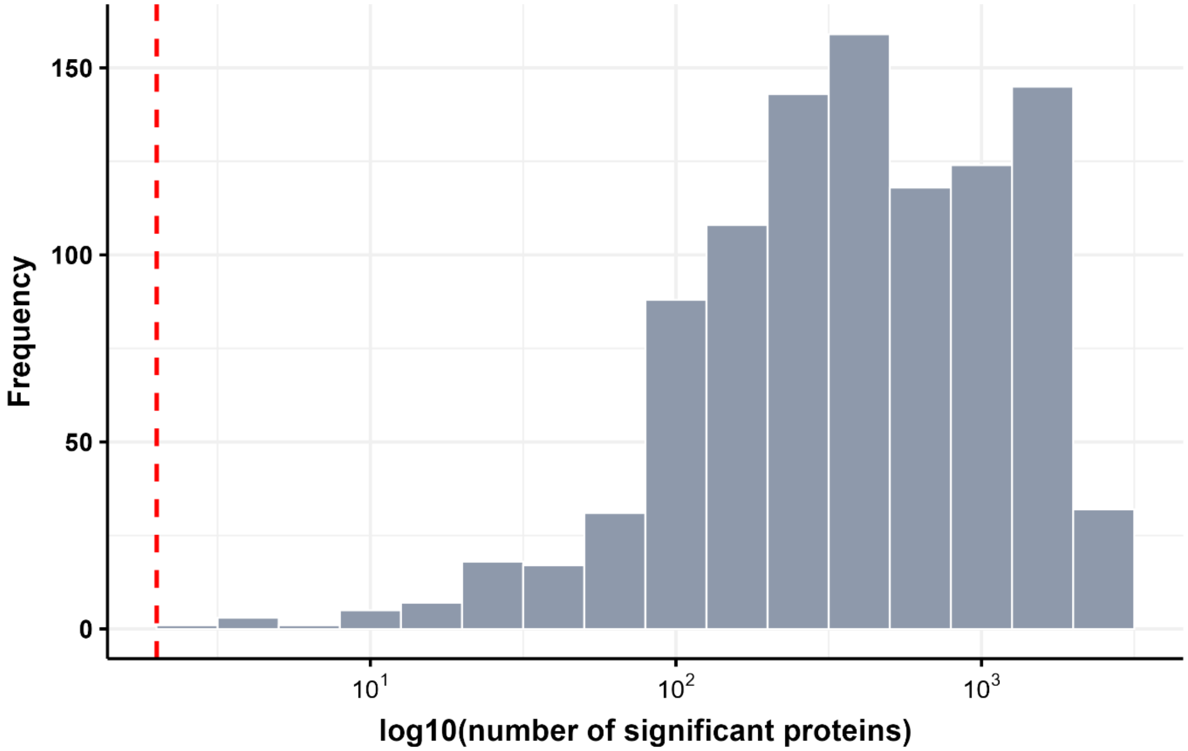

Figure S1: Frequency of 2 or fewer significant proteins with reduced sample size using full participant group

We assessed the likelihood of observing two or fewer significant proteins given the reduced sample size of 22 high neutralizers and 32 low neutralizers. We randomly sampled 22 participants from the high neutralizers and 32 participants from the low neutralizers from the full group of participants, including those with supplemental oxygen, with replacement for 1000 iterations. We then conducted differential protein analysis on each iteration. We recorded the number of proteins with an FDR<0.05 with each analysis, yielding a distribution used to estimate the likelihood of observing two or fewer proteins. Dashed vertical line at 2 proteins.

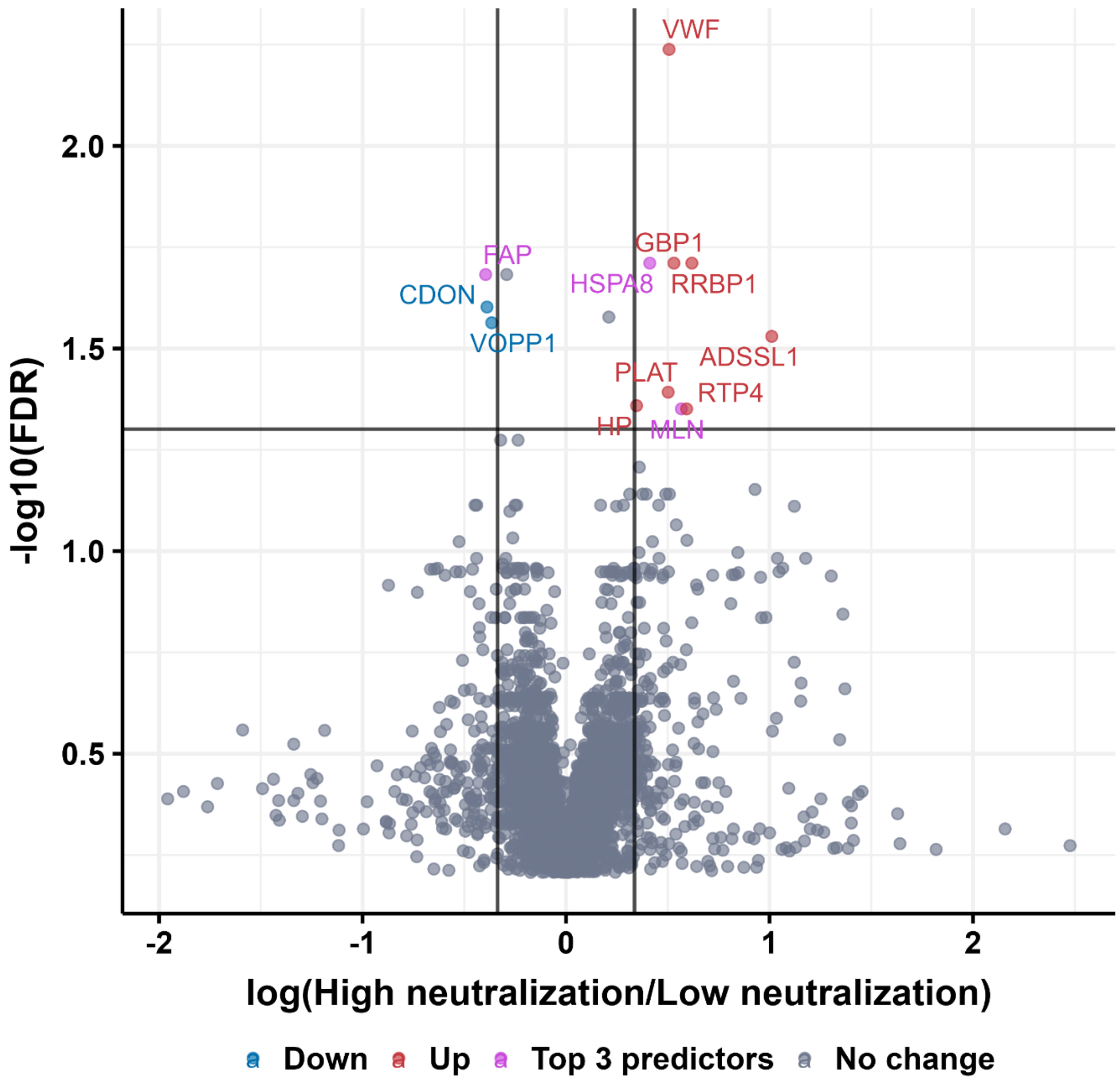

Figure S2: Significantly differentially expressed proteins in the training set

Volcano plots show fold change versus false discovery rate (FDR) values for each protein. The x-axis represents the log fold-change between the mean protein level values in the group of high versus low neutralizers or high versus low disease severity. Y-axis is the $-\log_{10}$ transformed FDR. The vertical lines indicate ±1.5-fold change and the horizontal line FDR=0.05. Significantly differentially expressed proteins are labeled in red (upregulated) or blue (downregulated). The proteins showing highest predictive value in the model are highlighted in purple.
