# Supplementary material for: Host proteins associated with strong neutralizing SARS-CoV-2 antibody responses in a South African cohort
Source: Commun Med (Lond). 2026 Feb 13;6:203. doi: 10.1038/s43856-026-01427-7 (PMC13066479; doi:10.1038/s43856-026-01427-7)
Supplement: Supplementary file 2 — Description of Additional Supplementary Files [file 43856_2026_1427_MOESM2_ESM.pdf]

Description of Additional Supplementary Files

Supplementary Data 1- Data used to generate all figures in this work
